# Retinal Thickness as a biomarker of cognitive impairment in bipolar disorder

**DOI:** 10.1192/j.eurpsy.2022.1054

**Published:** 2022-09-01

**Authors:** E. García-Corres, S. Alberich, L. Rementeria, I. Pérez-Landaluce, A.M. González-Pinto

**Affiliations:** 1 Osakidetza Basque Health Service, Araba University Hospital, Psychiatry, VITORIA-GASTEIZ, Spain; 2 BIOARABA, Neuroscience, VITORIA-GASTEIZ, Spain; 3 University of the Basque Country, Neuroscience, LEIOA, Spain; 4 CIBERSAM, Bipolar Disorder, Madrid, Spain

**Keywords:** cognition, biomarker, resilience, bipolar disorder

## Abstract

**Introduction:**

Ocular Coherence Tomography (OCT) to measure retinal thickness is the current method to observe neurological impairment in neurodegenerative diseases [1] and in mental disorders [2] due to the composition of the retina itself as an anatomic extension of the brain.There can be found some factors to improve the resilience like the years of study.

**Objectives:**

Our aim is to evaluate cognitive and clinical impairment in Bipolar Disorder and see the correlation to the retinal thinning.

**Methods:**

Twenty-seven patients diagnosed with Bipolar Disorder were assessed in the context of the FINEXT programme (3). Selective attention, executive functions and verbal memory were measured among other variables. Using the OCT technique, we measured the thickness of the ppRNFL, the RFNL, GCL and IPL layers in the macula in both eyes through several radial segments. Partial correlations were performed with Bonferroni correction (p≤0.006) adjusted for age and academic status except for the variable years of study which was adjusted for age.

**Results:**

Significant direct correlations were observed between: - The study-years and the thickness of the retina in the NO and RFNL. -Selective attention and GCL and RFNL layers. -Executive function and the GCL and IPL.

**Conclusions:**

We can observe some preliminar results showing a significant correlation between some layers of the retina, upper segments more frequently, and the outcomes of the neurocognitive assessment. We can see a relationship as well between years of study and the thickness of the Retinal Nerve Fibre Layer in the retina and optic nerve head, the axons of the neurons in the eye.

**Disclosure:**

No significant relationships.

